# Development of experimental pneumococcal vaccine for mucosal immunization

**DOI:** 10.1371/journal.pone.0218679

**Published:** 2019-06-28

**Authors:** Tatiana Gupalova, Galina Leontieva, Tatiana Kramskaya, Kornelya Grabovskaya, Eugenia Kuleshevich, Alexander Suvorov

**Affiliations:** 1 Department of Molecular Microbiology, Institute of Experimental Medicine, Saint-Petersburg, Russia; 2 Department of Fundamental Medicine and Medical Technologies, Faculty of Dentistry and Medical Technologies, Saint Petersburg State University, Saint-Petersburg, Russia; Universidad Nacional de la Plata, ARGENTINA

## Abstract

*Streptococcus pneumonia* is an important human pathogen that causes various severe diseases such as pneumonia, otitis and meningitis. Vaccination against *S*. *pneumoniae* is implemented in many developed countries. The presently used vaccines are safe, well tolerated but relatively expensive and require modification due to the immunological changes of the epidemic strains. This paper describes the development of a new pneumococcal vaccine candidate for immunization on mucosal surfaces. For this purpose the antigens of chimeric protein PSPF, previously suggested for an injectable *S*. *pneumoniae* vaccine, were expressed on the surface of the live probiotic strain *Enterococcus faecium* L3. Experiments on laboratory mice vaccinated with live bacteria demonstrated the appearance of the specific IgA and IgG which provide protection against the lethal *S*. *pneumoniae* infection.

## Introduction

*Streptococcus pneumoniae* is an important human pathogen which causes severe pneumonia, meningitis, otitis and often resulting in death. With the current deficit of active antibiotics due to the spread of antibiotic resistances, vaccination against *S*. *pneumoniae* has become the primary option for controlling this pathogen. Contemporary vaccines against *S*. *pneumoniae* based on targeting the capsule polysaccharides saved millions of lives but have some limitations related to the heterogeneity of capsule polysaccharides of the epidemic *S*. *pneumoniae* strains and short T-independent immunological memory [1].

This fact made it necessary to use the method of the conjugation of the capsular polysaccharides to toxoid molecules (which is not entirely safe) and requires a constant increase in the number of polysaccharides included in the vaccine due to the antigenic shift in the bacterial population (Red Queen Dynamics) [2,3]. All existing vaccines are injectable and so do not provide optimal mucosal protection at the major infection gate: the nasopharynx.

We have recently developed a protein-based chimeric vaccine PSPF for systemic immunization which is immunogenic and protective in mice [4]. The action of this vaccine on mucosal surfaces was substantially amplified by adding probiotic strains as adjuvants [5]. A chimeric protein, designated PSPF (Pneumococcus Surface Proteins and Flagellin), was constructed from conservative and immunogenic fragments of *S*. *pneumoniae* surface proteins: pneumococcal surface protein A (PspA), the surface protein Spr1875, pneumococcal surface adhesion (PsaA) and the Salmonella typhiurium flagellin terminal domains FliC1, with FliC2 functioning as adjuvant. PSPF was immunogenic in mice and induced protection from virulent *S*. *pneumoniae* strains (3, 6B, 14 and 19F). Anti-PSPF sera recognized subtypes of *S*. *pneumoniae* 6, 9, 19F, 6ABC, 9VA, 19A, 3, 34, 14, 9L [4, 5].

The present article is devoted to the creation of a probiotic strain expressing PSPF on a bacterial surface for use as a new vaccine for mucosal immunization.

## Materials and methods

### Making a chimeric protein from *E*. *faecium* L3 *d2* gene and a fragment of the *pspf* gene

Chromosomal DNA was isolated in order to use the probiotic strain *Enterococcus faecium* L3 chromosome as a template in a polymerase chain reaction (PCR). A plasmid *pspf* DNA was used as a template in order to amplify a portion of *pspf* gene as described in detail previously [4, 6].

DNA fragments corresponding to the fragments of the *d2* gene from *E*. *faecium* L3- encoding pili protein and the fragment of the *pspf* gene were amplified by PCR with Taq polymerase (AmpliTaq, Perkin-Elmer, Cetus, USA) using a thermocycler (BIO-RAD, USA). The oligonucleotide primers used for the reaction are listed in Table 1. The PCR program included denaturation at 94°C for 30 sec, primer annealing at 55°C for 1 min, and synthesis at 72°C for 1 min. This cycle was repeated 30 times, after which the mixture was incubated at 72°C for 10 minutes. Three fragments of DNA were obtained as a result of three separate reactions with the primers A1-B1 and C1- D1 for enterococcal DNA, and the primers E1 and F1 for *pspf* gene. Amplified DNA segments were isolated from the agarose using the QIAquick Gel Extraction Kit (Qiagen, USA).

**Table 1:** Oligonucleotide primers.

Primers	Direction	Nucleotide sequence from 5' to 3'
A1	forward	GC**TCTAGA**GCCGATGAGAGCAGCTGGTATTG
B1	reverse	AGGTCAGCCGGTAC**CATATG**CAATGCGCCATCATAGTTT
C1	forward	CGACAAGCTGGATAAA**CTCGAG**AAAGGTTCTGCGCGAGTGATAGAT
D1	reverse	CAACA**GGATCC**AAAGCATCGTTGG
E1	forward	**CATATG**GTACCGGCTGACCT
F1	reverse	TTT**CTCGAG**TTTATCCAGCTTGTCG
B2	forward	TGAGTGAACCACAGCCAGAA
Seq F	forward	GGACACCACAACCATCGAAG
Seq R	reverse	TAGTCCTCTTCTGCCTGCTG

The bold sections in the nucleotide sequences correspond to restriction sites.

### Preparation of a fusion gene *ent-pspf* and cloning

A hybrid DNA fragment was generated with primers A1 and D1 in PCR using the program described above, wherein the synthesis time at 72°C was increased to 2 minutes. DNA isolation and the analysis of the size of the amplified portion of the resulting DNA fragments were performed as described above. The cloning of the amplified DNA fragment was performed using plasmids pJET1.2, Clone JET PCR Cloning Kit. A ligation mixture was used to transform *E*. *coli* DH5α. The medium for selection of the transformants contained 100 μg/ml ampicillin. Hybrid (*ent-pspf*) DNA was subcloned into a suicidal plasmid pT7ermB with the gene of resistance to erythromycin. For this purpose, an amplification using the primers A1 and D1 was carried out. The PCR product and the plasmid pT7ermb were digested by enzymes BamHI and XbaI. The products of hydrolysis were separated by electrophoresis in 1% agarose gel and purified from the agarose using the set QIAquick Gel Extraction Kit (Qiagen, USA), then ligated and transformed into the *E*. *coli* DH5α heterologous system. The Luria Broth (LB) medium for selection of *E*. *coli* transformants contained 500 μg/ml of erythromycin. In order to check the construct, plasmid DNA *pent-pspf* was used as a template in a PCR with primers A1and B1, C1 and D1, A1 and F1, E1 and D1.

### Electroporation of enterococci

In the first step of the transformation process *Enterococcus faecium* L3 culture was cultivated in 3 ml of Todd-Hewitt broth (THB) (HiMedia, India) and grown overnight at 37°C; then, 1 ml of the culture was re-suspended in 50 ml of THB broth and grown to an optical density of 0.3 at 650 nm. After that, the culture was placed in ice and then washed three times in 20 ml of 10% glycerol at 4°C. The resulting bacterial pellet was suspended in 0.5 ml of sterile 10% glycerol solution and transferred into Eppendorf tubes, 50 μl per tube. After the DNA (300 ng) was added, the enterococci were electroporated in a cuvette with a 1 mm electrode spacing at 2100 V. The pulse duration was 4.5 milliseconds. After the current was discharged, 1 ml of THB was added to the cuvette, incubated for 1 hour and plated on selective media containing 10 μg/ml of erythromycin. Transformants were selected after 24 hours of cultivation on selective media with 5 μg/ml of erythromycin.

### Analysis of the nucleotide and amino acid sequences

DNA sequencing was performed by the research and production company Syntol. The amino acid sequence was determined based on the nucleotide sequence using the computer program ExPASy’s translate tool [6].

### Bacterial cultures

An *E*. *coli DH5α* strain was obtained from the strain collection of the Institute of Experimental Medicine and used as the recipient in the transformation. Bacteria were grown in LB medium at 37°C with constant shaking.

A culture of the original *E*. *faecium* L3 strain or genetically modified *E*. *faecium* L3-PSPF+ was grown in sterile THB (c 0.5% yeast extract) and incubated at 37^0^ C for 24 hours. Bacteria were washed three times by centrifugation at 3500 rpm for 20 minutes. The bacterial sediment was suspended in PBS to the desired concentration. The resulting suspension was used for vaccination of mice. LB agar (Lennox L agar, Thermo Fisher Scientific) and Enterococcus Differential Agar Base (TITG Agar Base) (Himedia, India), without antibiotic and with 2,5 μg/ml of erythromycin were used as a solid medium for cultivation, bacterial quantification, and identification of *E*. *faecium* L3 and erythromycin- resistant enterococcal transformants.

Serotype 3 *S*. *pneumoniae* clinical isolate (strain 73) was obtained from the collection of the Research Institute of Pediatric Infections (St. Petersburg, Russia). Pneumococci were cultured at 37°C and 5% CO2 for 18 hours in THB medium with 20% horse serum (Difco). Columbia agar with 5% defibrinated sheep blood and 10% horse serum was used as a solid medium.

Bacteria were washed three times by centrifugation at 3500 rpm for 20 minutes. The bacterial sediment was suspended in PBS to the desired concentration. The resulting suspension was used for infecting mice.

### Method of bacteria counting

10 μl of 10-fold sample dilutions were dropped on the surface of solid medium and incubated for 24 hours at 37° C and 5% CO2. The bacterial number was counted in each drop. By extrapolation, this number was used to calculate CFU (colony forming units) per milliliter of original sample.

### Ethics statement

All of the animal experiments were carried out under the guidelines of the “Rules of Laboratory Practice” of the Ministry of Health of the Russian Federation N° 708. The study was approved by the Local Ethics Committee for Animal Care and Use at the Institute of Experimental Medicine, Saint-Petersburg, Russia. Non-terminal procedures were performed under ether anesthesia. Animals were euthanized under ether anesthesia by cervical dislocation. The health status of the live vaccine- challenged mice was monitored and recorded once a day for ten days post-vaccination. No animal showed any signs of illness following vaccine strain infection. No animals died (without euthanasia) as a result of the experimental procedures.

### Immunization of mice with live probiotic vaccine

The 8- to-10-week-old female CBA mice were provided by the laboratory breeding nursery of the Russian Academy of Sciences (Rappolovo, Leningrad Region). Mice were housed in groups of twenty in 400×250×200 mm cages (Plastpolymer, Russia), maintained under standard conditions and given ten days to acclimate to the housing facility. All animals were housed in a special pathogen-free facility, and fed autoclaved food and water ad libitum. At the start of the experiments, the animals weighed (mean±SD) 17.0±2.0 grams. The immunogenic properties of the *E*. *faecium* L3-PSPF+ vaccine strain were tested for intranasal modes of administration. Mice were randomly distributed into two groups. A total volume of 20 μl of *E*. *faecium* L3-PSPF+ at a dose of 4 x 10^8^ CFU per mouse was administered through the nasal cavity between the left and right nares of mice (n = 20) on days 1, 2, 21, 22, 42 and 43 from the start of the experiment. The control group (n = 20) was vaccinated with the original *E*. *faecium* L3 strain in the same way.

Blood samples were taken from the submaxillary vein, and nasal lavages were obtained. For this purpose, mice were injected intraperitoneally with 0.1 ml of a 0.5% pilocarpine solution, and after 1 to 2 minutes, immediately after the onset of increased salivation, 50 μl of secretions was collected. The PMSF protease inhibitor was added to the samples for a final concentration of 1 mM.

There were no less than three days between blood or nasal sampling and infection.

### Specific immunoglobulins detection

Specific IgG and IgA levels were determined by ELISA in 96-well ELISA plates (Nunc) coated with the protein PSPF (2 μg/ml) overnight at 4°C. A series of twofold dilutions of the sample (100 μl) was added to duplicate wells and incubated for 1 h at 37°C. HRP-labeled goat anti-mouse IgA and IgG antibodies (Sigma) diluted in a blocking buffer according to the manufacturer’s instructions were added (100 μl/well). After incubation at 37°C for 1 hour, the plates were developed with TMB substrate (BD Bioscience) according to the manufacturer’s instructions. IgA and IgG antibody levels were expressed as the mean OD_450_ of appropriate dilution of sera from 10 mice per group ± SD.

### Study of the protective efficacy of vaccination

In order to investigate the specific protective effectiveness of the immune response, immune and control mice were infected with serotype 3 of *Streptococcus pneumoniae* (strain 73) intranasally at a dose of 6.0 x 10^4^ CFU/mouse. The effectiveness of immune protection was evaluated based on the pneumococcus burden in the lungs and mice mortality from the infection. Lung tissues were collected after 4 hours from 10 mice per group. The other 10 mice in each group were monitored for 10 days to determine the level of mortality.

Lung tissues were homogenized in PBS using a Retsch MM-400 ball vibratory mill. Serial 10-fold dilutions of homogenates were made in PBS and aliquots of the dilutions were plated on Columbia agar with 5% defibrinated sheep blood and 10% horse serum. The bacterial burden in CFU per organ was calculated and expressed as log_10_ according to the method of bacteria counting mentioned above.

### Statistical analysis

Data were processed using Statistica software, version 8.0. (StatSoft, USA). Means and standard errors of the means (SEM) were calculated to represent bacterial titers. The significance of differences between the two groups was determined using student’s test (analysis of bacterial burdens and antibody levels. Kaplan-Meier log rank test was used to analyze mortality data (p<0.05 was considered statistically significant).

## Results and discussion

**Fig 1:**
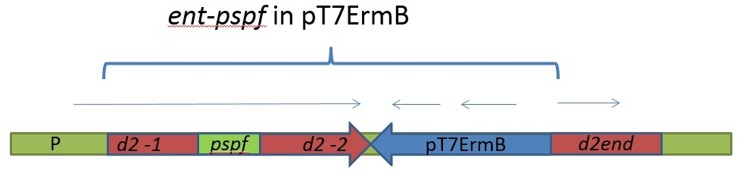
Integration scheme of the plasmid pT7ermB with the *ent-pspf* into the chromosome of the strain *E*. *faecium* L3. P is the promoter of the gene *d2; d2*-1 is a region of the *d2* gene encoding for N- terminal part of D2 protein; d2-2 is a region of the d2 gene encoding for central portion of D2 protein; *pspf* is *DNA* encoding PSPF chimeric protein; d2-end is the end of the *d2* gene encoding for the C terminus of D2 protein; pT7 ErmB is the integrative plasmid. Arrows correspond to the open reading frames in the integrated element. The entire integrated element *ent-pspf* with plasmid pT7ErmB is shown in brackets.

A chimeric construct with the genetic element *pspf* encoding for pneumococcal vaccine was inserted in frame into enterococcal gene *d2* by PCR, as described in the Material and Methods section (Fig 1). The resultant DNA *ent-pspf*, 1.8 kb in size, was cloned in *E*. *coli* plasmid pJET1.2. Later, *ent-pspf* was re-cloned in suicidal plasmid pT7ermB, which is unable to replicate in gram-positive bacteria.

The stages of cloning were tested by DNA analysis, and are presented in supplementary materials (S1–S3 Figs).

An integrative plasmid with *ent-pspf* DNA was used for the electroporation of the enterococcal strain *E*. *faecium* L3 as described in Materials and Methods. Erythromycin-resistant enterococcal transformants were tested for the presence of the insert of the plasmid into the genome by PCR and the corresponding DNA sequencing of one of the transformants (S4 and S5 Figs).

One PSPF+ positive enterococcal clone was selected, designated *E*. *faecium* L3 PSPF+, and used in a vaccine preparation for further study.

**Fig 2:**
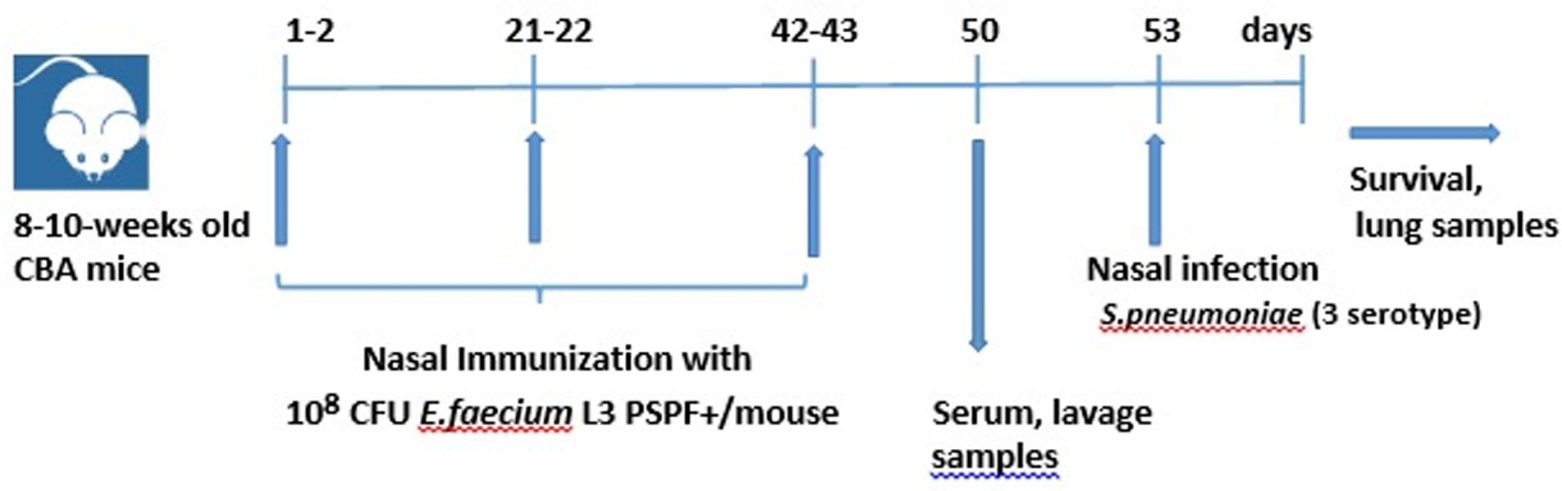
Immunological study and analysis of *E*. *faecium* L3 PSPF+ protective properties. General setup.

It was necessary to determine whether *E*. *faecium* L3 PSPF+ stimulated a PSPF-specific immune response after application through mucosa, and whether the immune response was sufficient to protect mice against pneumococcal infection. The experimental group of mice was vaccinated with live vaccine *E*. *faecium* L3 PSPF+ through the mucosal surfaces of the nose and upper respiratory tract. The control group was vaccinated with the original *E*. *faecium* L3 strain in the same way. Secretory and systemic immune responses were measured over the course of vaccination. The effectiveness of vaccination was evaluated by comparing the resistance of immune and control mice to pneumococcal infection. For this purpose, both groups of mice were infected intranasally with *S*. *pneumoniae* serotype 3. The bacterial burden was examined in the lungs and the mortality from infection was estimated within 10 days after infection. The general experimental setup is presented in Fig 2.

The vaccination course was conducted in three stages. Each stage included two daily nasal applications of enterococci.

It was necessary to find out whether enterococci colonized the mucous surfaces of the respiratory tract after vaccination.

For this purpose, on experiment days 3, 4, and 5 after the second vaccination the bacterial composition of the nasal lavages was determined by screening on selective medium of Enterococcus Differential Agar. Enterococci were not detected in nasal lavages. This indicated the lack of persistence or respiratory tract colonization by *E*. *faecium* in mice after the course of application.

**Fig 3:**
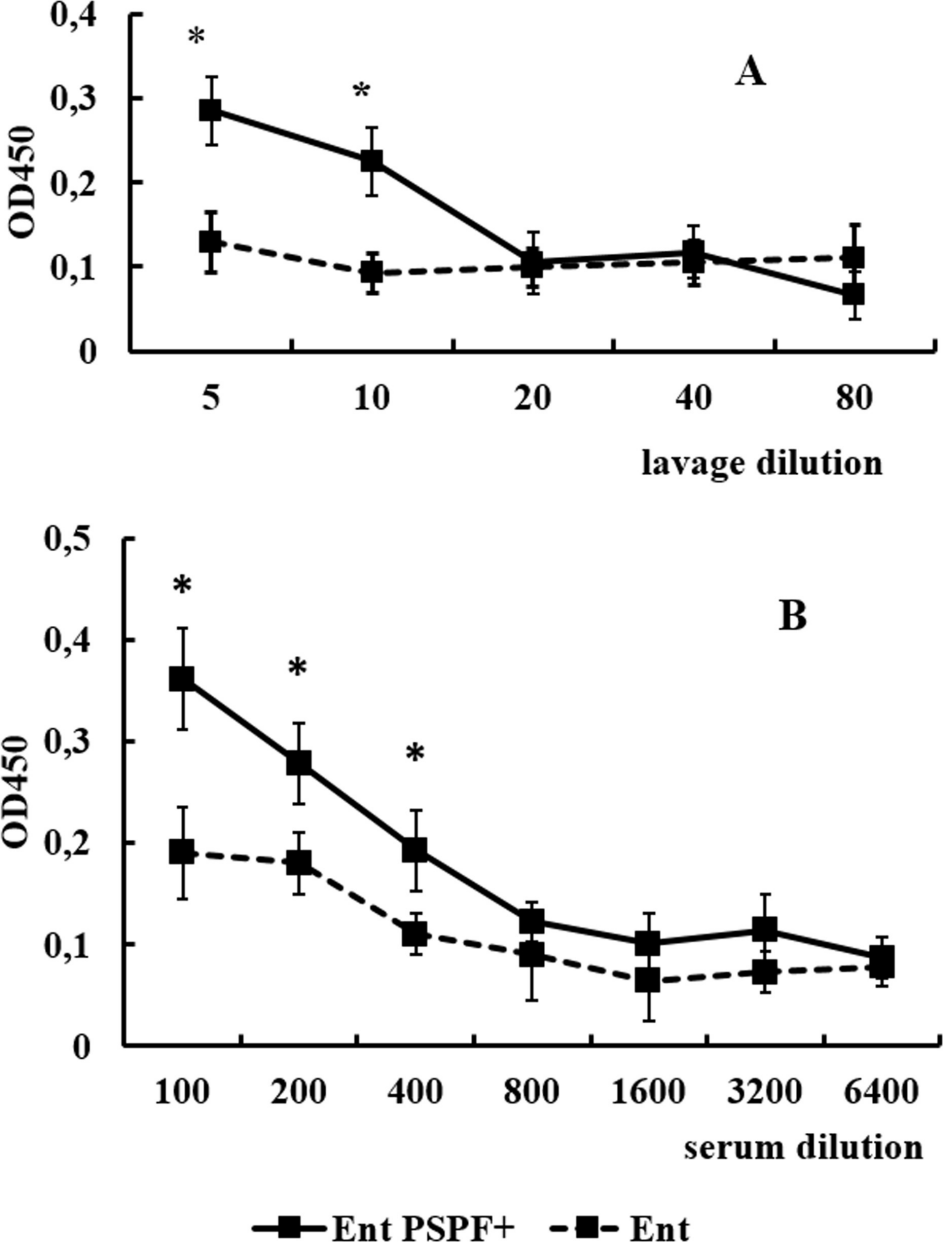
**(A, B). Anti-PSPF antibody levels in nasal lavages and serum samples after i.n. vaccination with live vaccine.** PSPF-specific total IgA (A) (n = 10 per group) and IgG (B) (n = 10 per group) antibodies were measured by ELISA on day 7 after the third round of vaccination. Each OD_450_ symbol represents the mean of 10 mice±SEM. The asterisk indicates values that are statistically significantly different (p≤0.05).

A week after the end of vaccination, the rate of PSPF-specific antibodies was measured in nasal lavages (Fig 3A) and serum (Fig 3B).

Intranasal mucosal immunization of mice with the PSPF-modified probiotics stimulated a specific immune response both in nasal lavages and in the serum. PSPF-specific secretory IgA were detected in nasal lavages (Fig 3A) and specific IgG antibodies was registered in serum in response to vaccination (Fig 3B).

**Fig 4:**
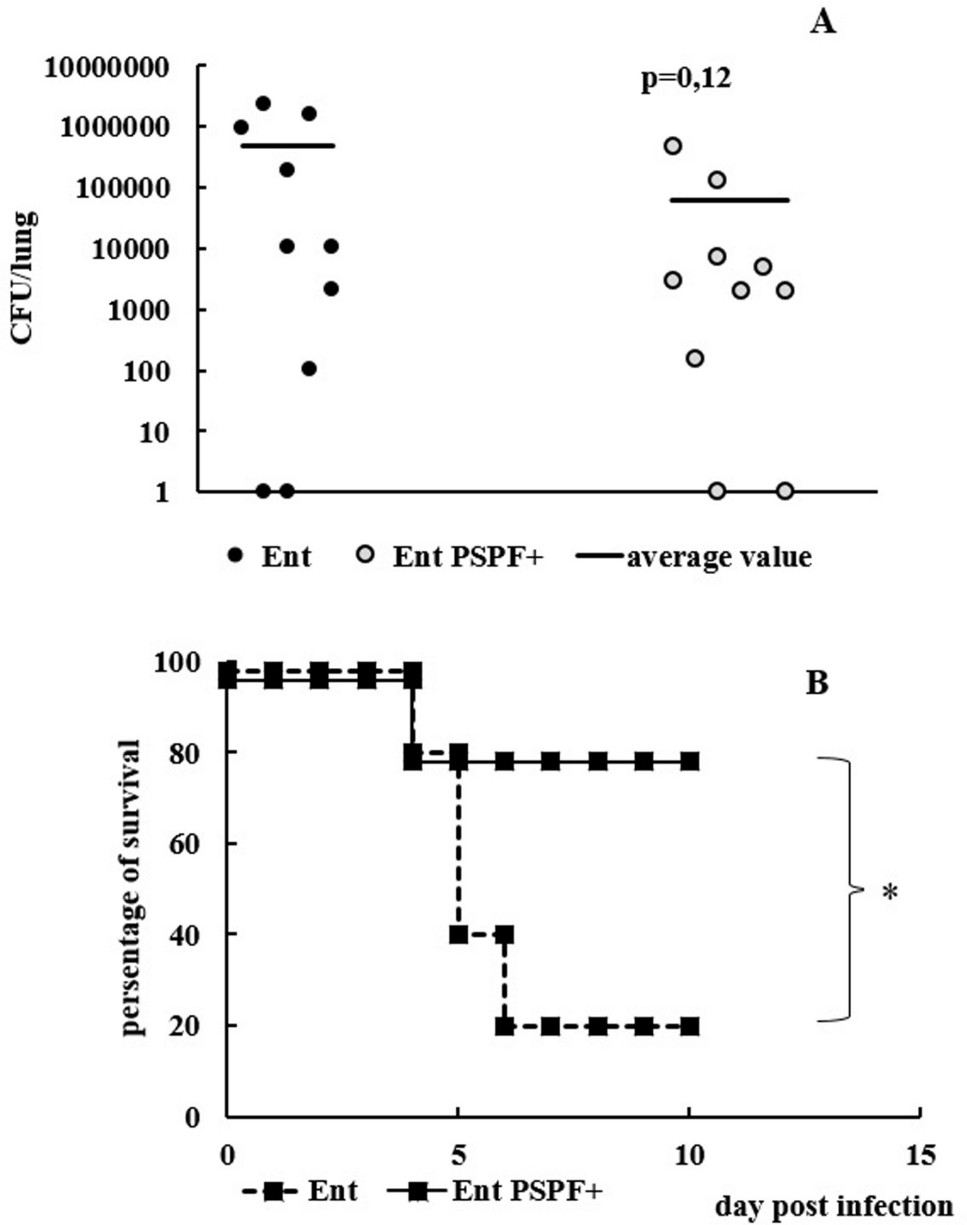
Protective efficacy of a specific immune response. Vaccinated and control mice (20 mice per group) were infected with *S*. *pneumoniae* strain 73 serotype 3. (A) The bacterial burden in lungs (CFU) was tested four hours after infection. The number of bacteria was calculated individually and expressed as CFU/lung. (B) The other 10 mice in each group were monitored for 10 days to determine the level of mortality. The asterisk indicates values that are statistically significantly different (p≤0.05).

To evaluate the protective efficacy of a specific immune response, vaccinated mice were infected by *Streptococcus pneumonia* strain 73 (serotype 3). The respiratory infection of immune mice was induced 10 days after the end of the vaccination courses. In order to assess the rate of protection, we checked the bacterial burden in CFU per lung 4 hours after infection (Fig 4A) and evaluated the mortality rate over the course of pneumococcal infection (Fig 4B). Mice administered the original strain of *E*. *faecium* L3 in PBS by an equivalent route served as a control.

Four hours after infection we found a downward trend (p = 0.12) in the number of bacteria in the lungs of the immune mice compared to the control (Fig 4A).

Mortality rates in the control and experimental groups (Fig 4B) differed significantly (p<0,05). Vaccinated mice were more resistant to lethal pneumococcal infection than control animals. The data indicated that the intranasal route of vaccination with chimeric probiotic live vaccine provided protection against pneumococcal infection of the respiratory tract in mice.

The present paper is a continuation of studies aimed at creating live vaccines with probiotic bacteria used as a vector for vaccine antigens. Previously, we developed a technology for the introduction of DNA constructs into genes that encode fimbrial proteins located on the surface of probiotic enterococci. For this purpose we had created and inserted into the enterococcus a chimeric fimbrial protein d2 containing the immunogenic fragment of the Bac streptococcal protein, and tested it as a possible protective group B streptococcal vaccine [6]. In the present paper, this method was applied for making a probiotic live vaccine against *S*. *pneumoniae*.

*S*. *pneumoniae* is medically a highly important pathogen, requiring vaccine prophylactics all over the world. Vaccines currently used in general practice have limitations including incomplete immunological coverage, lack of immunogenicity of capsular antigens, which needs to be enhanced by combining vaccine antigens with modified toxins, and high cost [7–9].

Another disadvantage of routine vaccination is the technique of vaccination. Pneumococcal vaccines like many others are administered parenterally with a syringe, and are not applied to the mucous surfaces of the nasopharyngeal tract: the natural gates of respiratory infections. In this vaccination procedure, the immune response is less consistent with the natural immunological process after pneumococcal infection compared to the mucosal vaccination method.

Live vaccines similar to the one generated in the present study are designed for the intranasal route of administration. It is known that the introduction of antigens through the mucosa of the upper respiratory tract is a convenient way to stimulate the local secretory and systemic humoral immune response [10]. Various adjuvants, including probiotic microorganisms, are used to enhance the immune response [11–15].

The synthesized live vaccine is a probiotic strain with a vaccine antigen on its surface. Thus, we tried to combine in one preparation an adjuvant and a specific polypeptide antigenic component of the vaccine.

The introduction of the peptide fragment into the bacterium can be a way to overcome the limitations of free peptide administration, which is important for low molecular weight peptides. Low molecular weight antigens are theoretically able to penetrate the brain through the blood-brain barrier [16]. This problem is not present if the alien molecule is part of a live vaccine based on a probiotic strain.

Another advantage of the suggested live bacterial vaccine based on enterococci is the absence of the active vaccine strain reproduction in the nasal or oral cavity, which might disrupt the natural consortium of microorganisms on the mucosa of the upper respiratory tract [17–19].

The designed enterococcus vaccine did not colonize the mucosa of experimental mice for more than 24 hours, despite the high and repeatedly administered infectious dose. After the first vaccination, enterococci in the epithelial barrier area are probably effectively controlled by the innate immune system. In the subsequent stages of vaccination, adaptive immune mechanisms also contribute to the elimination of enterococcus.

In addition, exogenous enterococci are rapidly replaced by a natural commensal flora that has effectively adapted to coexist with the macro organism due to a number of mechanisms. These include: a chronic immune response that constitutively maintains commensals at a reasonable distance from the host cells (i.e., physiological inflammation), a low immune responsiveness of the host towards commensals, and the active dampening of the immune response by commensals, as described in detail previously [20].

It can be assumed that the vaccine variant of enterococcus is not perceived as an obvious pathogen. On the contrary, under conditions of experimental dysbiosis, the introduction of *E*. *faecium* L3 has a pronounced therapeutic effect, helps to relieve inflammation of the gastrointestinal tract and restore normal microbiota [21].

At the same time, Enterococcus, which contains the epitopes corresponding to the virulence factors from *S*. *pneumoniae* in the pili, can trigger the signaling mechanisms of host cells in the same way as pathogenic bacteria.

The administration of *E*. *faecium* L3 PSPF+ to laboratory animals promoted stimulation of the secretory and systemic immune response. On day 50 from the start of vaccination, specific IgA were detected in the nasal lavages of mice, and specific IgG antibodies were found in the serum. Thus, the protein PSPF in the form of insertion into the pili stimulated local and systemic specific immune response, as did the free form of recombinant chimeric PSPF protein [4].

As described previously [22–24] for synthetic live vaccines that carry a foreign molecule, it is important how the specific antigenic determinants are localized in the carrier bacteria. It is now established that antigen-specific humoral immunity can increase significantly when antigens are exported either to the carrier surface or extracellularly into the surrounding milieu, rather than remaining in the cytoplasm.

The vaccine antigenic determinants of *E*. *faecium*- PSPF, localized on the surface of bacteria in the structure of the enterococcus surface pilus, were able to stimulate the immune response even in the absence of persistent colonization of bacteria in the mucous membrane.

The protective efficacy of the immune response was studied after intranasal infection of immune and control mice with a lethal dose of *S*. *S*. *pneumoniae* serotype 3. This serotype is far ahead of other pneumococcal serotypes in terms of capsule size, virulence, and the resulting mortality [25]. It was shown that 13- and 23-valent polysaccharide vaccines give the weakest protection against pneumococci of this serotype [26–27].

The bacterial burden in the lungs of immune and control mice was analyzed. In the present study, we evaluated the bacterial load in the lungs of mice four hours after the onset of infection. Such a relatively short period after the infection was chosen because, to our judgment, the neutralizing effect of existing specific IgA antibodies of the mucous membrane is noticeable at the earliest stages of infection. Mucosal immune responses represent the first barrier of defense against respiratory infections [28]. Immunoglobulin A (IgA) is the predominant Ig isotype in mucosal tissues [29], and its importance in the defense against respiratory agents has been repeatedly shown [30–33].

Four hours after infection, a decrease in pneumococcus number in the lungs of immune mice was recorded in comparison to the control. According to the data of Magda S. Jonczyket. al., at 24 h post- infection the level of bacteremia shows a lower correlation with the survival [34], but the bacterial load in tissues at the time of death is highly correlated with the survival time.

Subsequent monitoring of mice mortality for 10 days showed that pre-vaccination with a probiotic vaccine provided protection and a 60% reduction in mortality with respect to the control group of mice. Thus, intranasal administration of the vaccine strain *E*. *faecium* L3 PSPF+ to mice stimulated a specific secretory and systemic immune response that provided protection from infection with *S*. *pneumoniae* serotype 3. Pneumococcal PSPF contains three antigenic determinants corresponding to the conserved immunodominant regions of the three surface proteins of *S*. *pneumoniae*: PspA, PsaA, and SprI895. We have previously shown that serum IgG antibodies specific for PSPF is able to bind to the surface of pneumococci of a variety of serotypes, thus showing their wide specificity [4], and that mice immunized with PSPF are protected from infection by pneumococcal serotypes 6B, 14, 3, 19F[15]. Consequently, there is reason to believe that the protective specificity of the newly constructed live vaccine will be just as extensive. The latter, however, will be further investigated.

## Conclusions

Vaccination against *S*. *pneumoniae* is becoming a routine procedure in most developed countries. However, none of the contemporary vaccines against this important pathogen are specifically designed to generate an immune response against the pathogen at the gates of infection. We have recently developed a technology, which enables the expression of protective antigens in probiotics; we have constructed a probiotic strain that expresses a set of conservative pneumococcal antigens. Vaccination with live probiotics stimulated the generation of specific IgA and IgG, indicative of the development of a local and systemic immune response. Immunized mice were more resistant to the infection with type 3 *S*. *pneumoniae* strain 73 than the control group. These results allow us to suppose that the recombinant probiotic strain with the chimeric vaccine might be considered as a possible vaccine prototype against *S*. *pneumoniae*. The versatility of the vaccine for various pneumococcal serotypes requires further research.

## Supporting information

S1 Fig 1% agarose gel electrophoresis pattern of the amplified DNA fragments used for making a chimeric construct. 1 –the PCR product with the primers A1 and B1; 2 –the PCR product with the primers C1 and D1; 3 –the PCR product with the primers E1 and F1; 4–100 bp Ladder DNA marker (100–3000 bp).
(PDF)

S2 Fig 1% agarose gel electrophoresis of the amplified fused DNA fragment. 1—PCR Product («fused» gene) with the primers A1 and D1; 2–100 bp Ladder DNA marker (100–3000 bp).
(PDF)

S3 Fig 1% agarose gel electrophoresis of the amplified plasmid pent-*pspf*. 1 –the PCR product with the primers A1 and B1; 2 –the PCR product with the primers C1 and D1; 3 –the PCR product with the primers A1 and F1; 4 –the PCR product with the primers E1 and D1; 5–100 bp Ladder DNA marker (100–3000 bp).
(PDF)

S4 Fig 1% agarose gel electrophoresis pattern of the amplified DNA fragments. 1–100 bp Ladder DNA marker (100–3000 bp); 2 –the PCR product of clone 1 with the primers SeqF and SeqR; 3 –the PCR product of clone 2 with the primers SeqF and SeqR; 4 –the PCR product of plasmid DNA pent-*pspf* with the primers SeqF and SeqR.
(PDF)

S5 Fig Nucleotide sequence of *E.faecium* L3 obtained after plasmid pent-*pspf* DNA integration. (PDF)
