# Supplementary figures and images for: Development of experimental pneumococcal vaccine for mucosal immunization
Source: PLoS One. 2019 Jun 28;14(6):e0218679. doi: 10.1371/journal.pone.0218679 (PMC6599147; doi:10.1371/journal.pone.0218679)

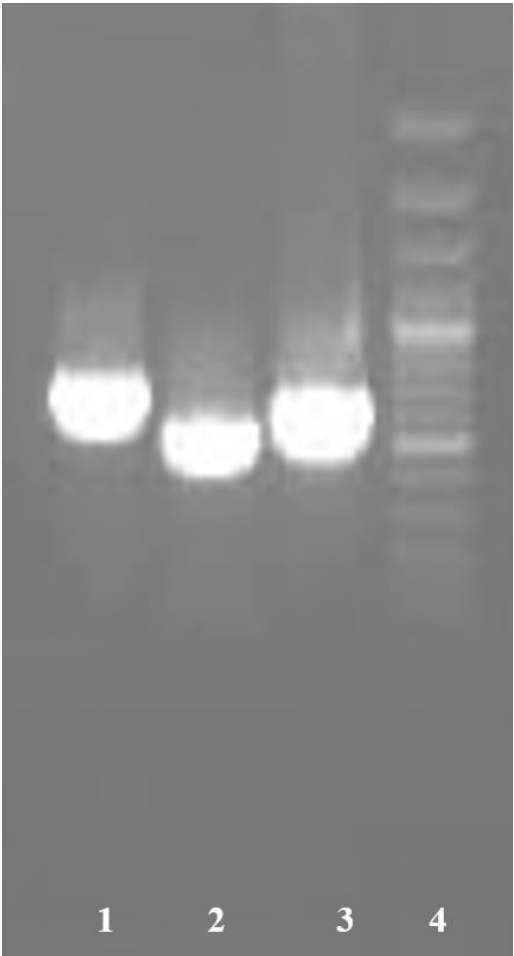

Supplement: S1 Fig — 1 –the PCR product with the primers A1 and B1; 2 –the PCR product with the primers C1 and D1; 3 –the PCR product with the primers E1 and F1; 4–100 bp Ladder DNA marker (100–3000 bp). (PDF) [file pone.0218679.s001.pdf]

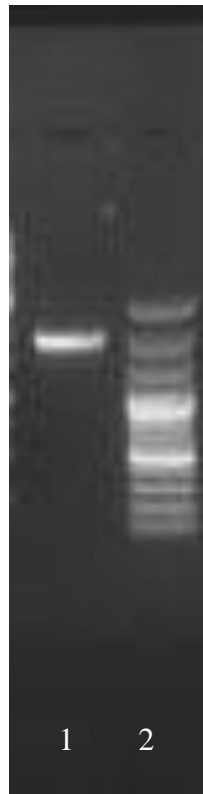

Supplement: S2 Fig — 1—PCR Product («fused» gene) with the primers A1 and D1; 2–100 bp Ladder DNA marker (100–3000 bp). (PDF) [file pone.0218679.s002.pdf]

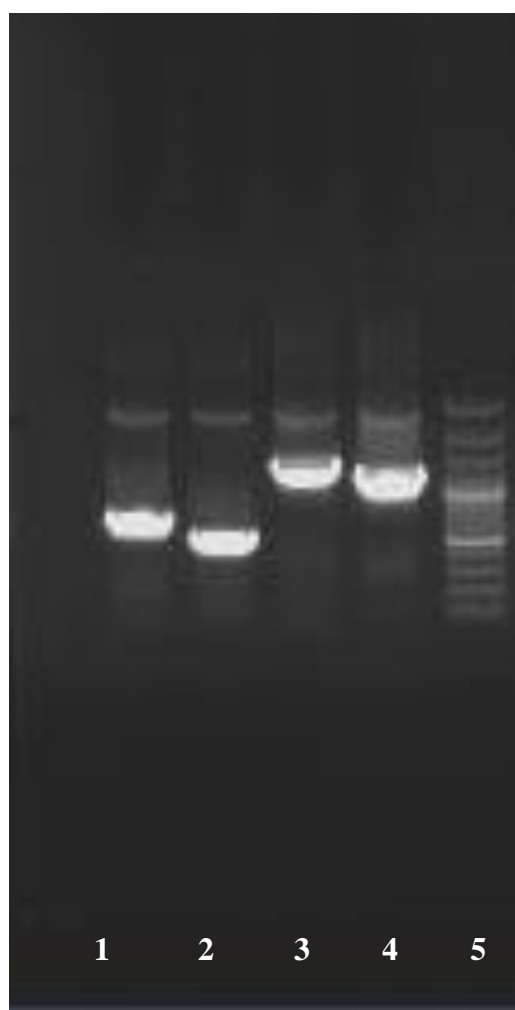

Supplement: S3 Fig — 1 –the PCR product with the primers A1 and B1; 2 –the PCR product with the primers C1 and D1; 3 –the PCR product with the primers A1 and F1; 4 –the PCR product with the primers E1 and D1; 5–100 bp Ladder DNA marker (100–3000 bp). (PDF) [file pone.0218679.s003.pdf]

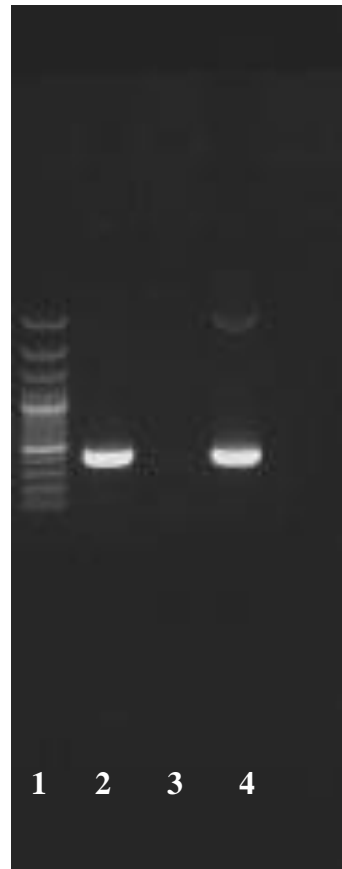

Supplement: S4 Fig — 1–100 bp Ladder DNA marker (100–3000 bp); 2 –the PCR product of clone 1 with the primers SeqF and SeqR; 3 –the PCR product of clone 2 with the primers SeqF and SeqR; 4 –the PCR product of plasmid DNA pent-pspf with the primers SeqF and SeqR. (PDF) [file pone.0218679.s004.pdf]
