# Supplementary material for: Development of experimental pneumococcal vaccine for mucosal immunization
Source: PLoS One. 2019 Jun 28;14(6):e0218679. doi: 10.1371/journal.pone.0218679 (PMC6599147; doi:10.1371/journal.pone.0218679)
Supplement: S5 Fig — (PDF) [file pone.0218679.s005.pdf]

primer B2 *d2-1*

TGAGTGAACCACAGCCAGAAATTAATTCAAAAATGAGATCGATGAGAGCAGCTGGT

ATTGAGTTGAATGATACATTTCTATCTATTTACAGTTTAAATGGACAGTATCAGCA

ACGTGTGTCTTGGTATAATGACAATAATGAATCTGTCGGTGAACGTAATATTGATA

TGAGAGAATTTGTTGGGTATGAAAAAATGGGTAGCTTACCTTATTTTGTCACAACA

GATACAGCATGTGCAGAATACAAAGCTCCTGCGTTATCAACAAACAATTTAACTTC

AAAAGTAGTGGGAGGACGTGCAGAAAAGGCTTATAGCTCGAATGATCATTTCACCG

ATGTTGTAGGAGCTGATACTTATCACAGAAGTGGTGTAACGTATACGCTTCAAGGC

GCTTCCCCAACATTCATGATTGGCGCAAATACGAATAGTATGATGTTTAGCTTTGA

TACTGCATTGCTATGGACACCACAACCATCGAAGCCTACAAAAGAAGTGTTTAACA

AAGCTAATACTGAAGAGGCAGCACACAATATTGACAAAAAAGTGATTCCACAAGGA

TCAGATGTTTACTATCATATTCATCAAAAGTTTGATGCATTAACAGTCAACACAAT

GAACAAATACAAATCATTTAAAATCACTGATACCTTTGACAGCAAAAATTTTGATA

pspf

TGGTATCGGATGGGAAAAACTATGATGGCGCATTGCATATGGTACCGGCTGACCTG

AAAAAAGCTGTTAACGAACCAGAAAAGCCGGCAGAAGAGGAACCGGAAAATCCGGC

CCCGGCGCCGAAGCCGGCGCCAGCTCCGCAGCCGGAAAAACCGGCCCCTGCTCCGG

primer SeqR

CTCCGAAACCGGAAAAATCTGCTGAC**CAGCAGGCAGAAGAGGACTA**
